# A Hybrid Multi-Scale Phase-Correlation Framework for Subpixel Registration of Multi-Temporal Very-High-Resolution Remote Sensing Images

**DOI:** 10.3390/jimaging12080347

**Published:** 2026-08-01

**Authors:** Laila Rasmy, Imane Sebari, Mohamed Ettarid

**Affiliations:** 1 Royal Center for Remote Sensing (CRTS), Rabat 10000, Morocco; 2 Morocco School of Geomatics Sciences and Surveying Engineering, Hassan II Institute of Agronomy and Veterinary Medicine, Rabat 10101, Morocco; i.sebari@iav.ac.ma (I.S.); m.ettarid@iav.ac.ma (M.E.)

**Keywords:** image registration, phase correlation, multi-scale methods, Gaussian pyramid, Simoncelli pyramid, sub-pixel accuracy, paraboloid fitting

## Abstract

This paper proposes a hybrid phase-correlation framework with a new multiscale detector, Fusion. Using a combination of FAST and Shi–Tomasi keypoints, followed by a probabilistic Hough transform and Canny edge detection, this detector improves repeatability. In addition, due to the limited ability of standard phase correlation to handle large geometric displacements, a complementary strategy is required to achieve sub-pixel matching precision. First, corners are extracted from both reference and sensed images using the Fusion detector. Corresponding points are then identified through coarse-to-fine phase correlation across a Gaussian pyramid. At each level, phase correlation yields an initial displacement, which is refined to sub-pixel accuracy using 1D parabolic fitting and propagated upward through the pyramid to obtain the final displacement. The proposed approach is evaluated using Pleiades and Sentinel-2 satellite images. Compared with the Scale-Invariant Feature Transform (SIFT)-based method and the detector-free Local Feature Transformer (LoFTR), the proposed framework achieves an RMSE below 0.2 and 0.4 pixels for Sentinel-2 and Pleiades imagery, respectively. Moreover, the results of the optimization analysis have revealed that shows that 2D paraboloid fitting combined achieves the lowest registration error of 0.010 pixels and the highest inlier ratio of 40.6%. The proposed approach achieves sub-pixel accuracy in the presence of noise and produces large numbers of correct matching points across different image resolutions.

## 1. Introduction

Accurate registration of multi-temporal remote sensing images is essential for a wide range of Earth observation applications, including change detection, image fusion, and environmental monitoring [1]. Its objective is to geometrically align images of the same scene acquired at different dates, from different viewpoints, or by different sensors. However, differences in imaging geometry and terrain relief introduce geometric and radiometric distortions that become particularly challenging in very-high-resolution (VHR) satellite imagery, where terrain-induced displacements generates locally varying geometric deformations that are difficult to model using conventional global transformation models [2]. Consequently, sub-pixel registration accuracy is essential to ensure that detected differences reflect actual physical changes rather than artifacts caused by geometric misalignment.

Existing image registration methods can generally be categorized into feature-based, area-based, and hybrid approaches. Feature-based methods establish correspondences by detecting, describing, and matching salient image primitives. Classical detectors such as Harris, Shi–Tomasi, and the Scale-Invariant Feature Transform (SIFT) have been widely adopted because of their computational efficiency and robustness to moderate geometric transformations. Nevertheless, their performance strongly depends on the repeatability, localization accuracy, and spatial distribution of the detected features. In particular, viewpoint changes, local terrain-induced distortions, and radiometric variations significantly reduce the reliability of feature matching. Moreover, an inherent trade-off exists between feature localization accuracy and repeatability, while maintaining stable feature extraction under varying illumination, atmospheric conditions, and sensor noise remains a challenging problem [3,4,5].

Area-based methods estimate image transformations directly from image intensities by maximizing similarity measures over local neighborhoods. Among these techniques, phase correlation has become one of the most widely used frequency-domain approaches because of its computational efficiency and robustness to global illumination and contrast variations. However, conventional phase-correlation methods assume locally consistent image structures and are therefore sensitive to nonlinear geometric distortions, relief displacement, large viewpoint differences, and heterogeneous radiometric conditions. Furthermore, exhaustive similarity evaluation over large search regions may considerably increase computational cost, particularly for VHR satellite images.

To overcome the limitations of purely feature-based and area-based methods, hybrid registration approaches have received increasing attention. These methods combine the complementary strengths of geometric primitives for robust correspondence initialization and area-based similarity measures for local refinement, thereby improving both registration robustness and sub-pixel accuracy [6,7]. Multi-scale representations have further enhanced these approaches by progressively estimating transformations from coarse to fine image resolutions, reducing sensitivity to noise, local ambiguities, and large geometric displacements.

Recent advances in deep learning have considerably improved image registration by learning robust feature representations directly from data [8,9,10]. Early learning-based methods relied on Siamese neural networks to learn feature similarity from image pairs [11]. Subsequently, self-supervised feature extraction methods enabled the joint learning of keypoint detection and feature description [12], while graph neural network-based frameworks improved correspondence estimation by modeling the spatial relationships between candidate matches. More recently, transformer-based architectures [13,14], have demonstrated remarkable performance by establishing dense correspondences without relying on explicit keypoint detection, substantially improving robustness to geometric distortions and radiometric variations.

In parallel, hybrid learning-based registration frameworks have extended classical phase correlation by integrating learned feature representations and spectral-domain modeling [15,16,17,18], improving robustness to multimodal imagery and complex geometric deformations.

Multi-scale representations remain a key component of both classical and learning-based registration methods. Hierarchical coarse-to-fine strategies improve convergence by progressively refining transformation estimates across multiple resolution levels, thereby increasing robustness to large displacements, scale changes, and local geometric distortions. Recent learning-based architectures further confirm the effectiveness of multi-scale processing for dense correspondence estimation and complex geometric transformations [18,19,20].

Despite these significant advances, several challenges remain. Learning-based methods generally require large annotated datasets and substantial computational resources, while their performance may deteriorate when applied to imaging conditions that differ from those encountered during training. Conversely, classical feature-based and frequency-domain methods remain computationally efficient but often struggle with complex local deformations, and heterogeneous radiometric conditions in VHR imagery. In addition, few existing methods effectively integrate robust feature detection, adaptive multi-scale processing, and sub-pixel phase-correlation refinement within a unified registration framework.

To address these limitations, this paper proposes a hybrid multi-scale registration framework that combines feature detection with local phase correlation and sub-pixel refinement to improve correspondence reliability and geometric registration accuracy for multi-temporal VHR satellite imagery under radiometric and geometric variations. By relying on analytical rather than learned representations, the proposed framework avoids the need for large annotated training datasets while maintaining computational efficiency.

This work extends the methodology introduced by Rasmy et al. (2021) through the following methodological enhancements [21]:

- Multi-scale coarse-to-fine registration: A Gaussian pyramid strategy is incorporated to improve robustness to large displacements, varying acquisition conditions, and terrain-induced geometric distortions.
- Multi-scale sub-pixel refinement: A sub-pixel estimation stage is integrated at each pyramid level with the aim of improving localization accuracy while maintaining consistency across scales and limiting error propagation.
- Fusion feature detector: A Fusion feature detector is proposed to provide a more suitable distribution of feature points and to support stable matching and geometric consistency.

In addition, seven sub-pixel estimation approaches, including iterative, spatial-domain, and frequency-domain techniques, are systematically evaluated to identify an appropriate configuration for high-precision image registration. These components are integrated into an extended hybrid co-registration framework that combines multi-scale processing, the Fusion detector, and sub-pixel refinement within a unified registration pipeline for multi-temporal VHR remote sensing image registration.

The proposed method is presented in Section 3, followed by the experimental results and discussion in Section 4. The conclusions are presented in Section 5.

## 2. Brief Overview of Related Work

This section reviews the main components of the proposed framework, with particular emphasis on phase correlation and pyramid-based multi-scale methods. Registration has been addressed using spatial and frequency-domain techniques. Spatial-domain methods estimate geometric transformations from local image intensities or local features, whereas frequency-domain approaches exploit spectral representations to recover image displacements efficiently. Although these techniques have achieved satisfactory performance in many applications, their effectiveness often degrades when applied to very VHR remote sensing imagery, where significant radiometric variations, relief-induced distortions, multimodal characteristics, and large geometric discrepancies are common. To improve robustness under these challenging conditions, coarse-to-fine strategies have become a standard paradigm, enabling the progressive estimation of image displacements from low to high spatial resolutions while reducing the risk of local minima. Within this context, phase correlation has remained a fundamental frequency-domain technique because of its computational efficiency and robustness to noise and uniform illumination changes. Recent advances have further extended its capabilities by integrating deep learning, adaptive spectral representations, and hybrid correlation models, substantially improving its performance for multimodal and large-displacement registration tasks.

### 2.1. Phase Correlation Methods

By exploiting the linear phase relationship between two images in the Fourier domain, phase correlation provides reliable estimation of global translational displacement. However, the classical phase correlation formulation is inherently limited to rigid translation and typically achieves only pixel-level accuracy, making it insufficient for high-resolution, multi-temporal, or multi-sensor remote sensing applications. To obtain sub-pixel accuracy, different refinement strategies have been developed in both the spatial and frequency domains.

In the spatial domain, the correlation peak is refined after the inverse Fourier transform using interpolation or curve fitting, including polynomial and Gaussian models [22], cubic splines [23], or sinc-based formulations [24,25]. Although these methods are computationally efficient, their accuracy may deteriorate in the presence of noise or boundary artifacts. Frequency-domain methods, in contrast, estimate the shift directly from phase differences without returning to the spatial domain. Representative approaches include plane fitting [26,27], line fitting [28], sawtooth models [29,30], and optimization-based techniques [31,32], which generally provide greater robustness and consistency under challenging imaging conditions.

While these approaches improve the performance of the classical phase-correlation framework, they still rely on predefined analytical models. To overcome these limitations, recent research has explored hybrid registration frameworks that combine the complementary strengths of phase correlation and feature-based techniques. In these methods, local feature detectors and descriptors provide reliable correspondences under geometric distortions, while phase correlation is employed to estimate accurate local displacements and achieve sub-pixel refinement. Such hybrid strategies improve robustness to scale changes, viewpoint variations, and multimodal image characteristics while preserving the computational efficiency of frequency-domain registration [21,32].

More recently, hybrid learning-based registration frameworks have emerged, integrating the mathematical foundations of phase correlation with learned feature representations to overcome the limitations of conventional Fourier-based formulations. Neural Phase Correlation proposed by Reynolds (2026) generalizes the classical phase-correlation framework by replacing the fixed Fourier basis with learnable filter pairs [16]. Recent advances have further extended frequency-domain registration through learning-based spectral representations [15,18]. In parallel, Peng et al. extend classical phase correlation by integrating multimodal local self-correlation descriptors, thereby improving robustness to nonlinear radiometric variations in heterogeneous image modalities [17].

These developments reflect the growing adoption of hybrid registration frameworks that combine the mathematical rigor and computational efficiency of Fourier-based methods with the robustness of feature-based techniques and deep learning to improve robustness under multimodal imaging conditions and complex geometric deformations.

### 2.2. Pyramid-Based Methods

Multi-scale representations help handle scale changes and improve robustness. These methods create multi-resolution representations, supporting coarse-to-fine analysis for better robustness to scale changes and distortions [20,33,34]. This coarse-to-fine approach reduces noise sensitivity and local ambiguities [35,36,37]. Several studies have further combined representations with feature-based or correlation-based techniques to improve correspondence reliability and registration accuracy under large geometric and radiometric variations [38]. Early work introduced hierarchical correlation with progressive refinement, which improves transformation estimation, especially when distortions are large [39,40,41,42]. Other approaches further enhanced robustness by adaptively selecting pyramid scales and correlation window sizes [19,43].

More recent approaches have focused on adaptive and hybrid multi-scale strategies. Dematteis et al. (2022) proposed a grid-based correlation method that progressively refines node spacing and correlation window sizes to achieve accurate multi-date image registration [19]. Li et al. (2022) combined phase correlation with iterative pyramid decomposition based on low-pass filtering and subsampling, improving the robustness and precision of satellite image registration [8]. Wavelet-based multi-scale representations have also been investigated. Moigne et al. (2002) showed that Simoncelli band-pass pyramids provide higher sub-pixel registration accuracy, whereas steerable low-pass pyramids offer greater stability [44]. More recently, learning-based registration frameworks have adopted hierarchical coarse-to-fine strategies, confirming the effectiveness of multi-scale representations for dense correspondence estimation and large geometric deformations [18,20].

Pyramid-based and coarse-to-fine methods remain an effective solution for image registration, especially when you combine them with correlation-based approaches.

## 3. Materials and Methods

Existing methods have limitations, and our previous work brought some improvements. Building on both, we now present our hybrid image registration framework. It aims to align VHR satellite images accurately and robustly. Our method combines feature detection, phase correlation, and sub-pixel refinement within a multi-scale framework. Instead of working at a single scale, we use a hierarchical coarse-to-fine strategy with multi-scale displacement accumulation. A locally adaptive sub-pixel refinement scheme is also integrated into the process to keep displacement estimation consistent from coarse initialization to fine-scale correction. The algorithm can handle large geometric changes, radiometric differences, and sub-pixel alignment in a unified framework. All experiments were conducted on a Windows 10 (64-bit) desktop with Python 3.12 and MATLAB R2018b (The MathWorks, Inc., Natick, MA, USA). The local desktop used for development and testing was a 64-bit Windows 10 computer equipped with an Intel® Core™ i7-10510U processor (Intel Corporation, Santa Clara, CA, USA), an NVIDIA Quadro P520 GPU (NVIDIA Corporation, Santa Clara, CA, USA), and 16 GB of RAM.

### 3.1. Overall Framework Workflow

**Figure 1:**
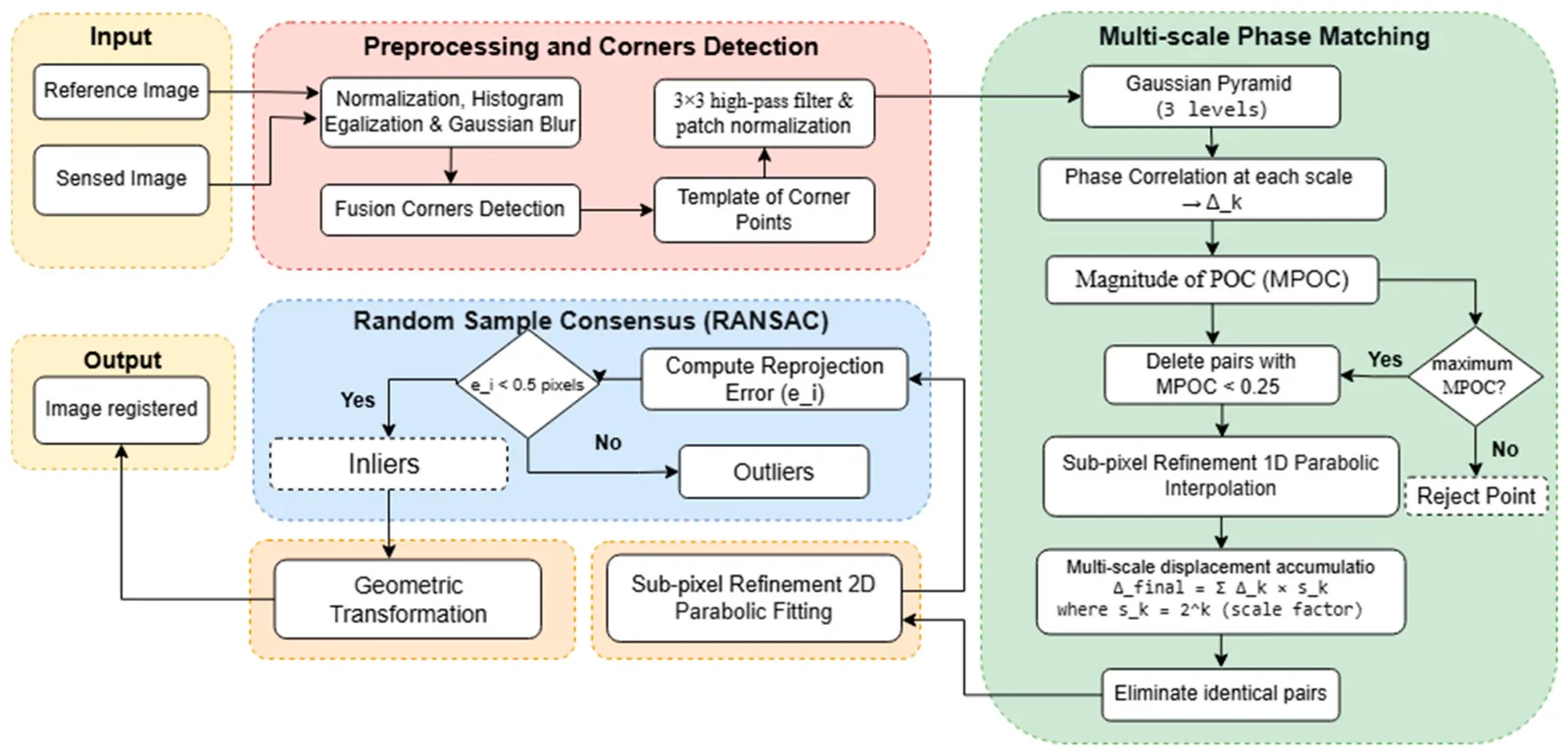
The workflow of our hybrid multi-scale image registration framework. It combines phase correlation, sub-pixel refinement, and RANSAC-based geometric transformation estimation.

Our hybrid sub-pixel registration algorithm works in several steps (see Figure 1). First, we preprocess both the reference and target images to improve robustness and reduce radiometric differences. Then, we extract interest points from each tile using a corner detector. Phase-only correlation is used to find matches, where the highest peak indicates the match location and its position provide the displacement. We then refine the displacement hierarchically, from coarse to fine resolution. The final transformation parameters are refined using least-squares optimization over the remaining inliers.

### 3.2. Preprocessing

We convert each image to grayscale and scale it to the 0–255 range. Then we apply histogram equalization and a Gaussian blur with a 3 × 3 kernel. For Harris-based detection, we also use CLAHE to enhance local contrast, especially useful in heterogeneous terrain. For each patch extracted, we apply a 3 × 3 high-pass filter to reduce illumination variations and enhance gradient information. Then, we normalize each patch to zero mean and unit variance to ensure radiometric consistency between reference and target images.

### 3.3. Interest Point Detection

Phase correlation can provide a dense displacement field at the pixel level, but its accuracy drops in low-texture regions or repetitive patterns. To improve robustness, we first extract a set of reliable anchor points to guide the motion field refinement. We use a multi-detector strategy to ensure the features are complementary and stable. Feature points are detected independently in both images using five detectors: Harris_Py (Python), Harris_Mat (MATLAB R2018b (The MathWorks, Inc., Natick, MA, USA)), Fusion, SIFT, and Shi–Tomasi.

Our Fusion detector is designed for VHR imagery. It combines FAST and Shi–Tomasi interest points at multiple scales. At each scale, FAST and Shi–Tomasi detectors are applied simultaneously to extract interest points, followed by edge detection using Canny and line segment detection using the probabilistic Hough transform. Only Shi–Tomasi points located within 3 pixels of detected line segments are retained. We process all the scales, then the retained points are then merged with FAST keypoints. Finally, duplicates are removed and the points are resampled to the original resolution.

FAST gives dense and fast keypoint extraction. Shi–Tomasi gives better stability and repeatability. Toobtainher, they balance feature density and robustness. Then we use a Hough line filter to keep only points that lie on strong geometric structures. This further improves robustness. With this multi-scale approach, we get keypoints that are dense but also geometrically meaningful—well suited for the registration steps that follow.

### 3.4. Patch Construction and Multi-Scale Pyramid Representation

We tested Simoncelli, Gaussian, and Laplacian pyramids on VHR multi-temporal datasets to identify the most suitable multi-scale representation for our hybrid registration framework. the proposed framework employs a three-level Gaussian pyramid with scales [1.0, 0.5, 0.25], where each level is generated by Gaussian filtering followed by dyadic downsampling. Registration is performed in a coarse-to-fine manner, starting from the coarsest level and progressively refining the alignment at higher resolutions.

The Gaussian pyramid offers the most robust and efficient foundation for hierarchical phase-correlation registration. It detects large displacements while keeping subpixel accuracy at the finest level. That makes it well suited for large-scale VHR remote sensing, where both accuracy and computational efficiency are critical.

### 3.5. Candidate Matching via Spatial Indexing

We implement an efficient spatial indexing strategy to reduce the search space while maintaining high matching accuracy. To establish point correspondences between the two images, we split the image into cells. Each cell is the size of our search radius $R_{s}=15$ pixels. Then we only match points in the same cell and its 3 × 3 neighboring cells. The complexity is reduced from $O\left(N^{2}\right)$ to $O\left(N\right)$ . For each candidate pair, we extract 16 × 16 pixel patches and check how similar they are with coarse-to-fine phase correlation.

### 3.6. Phase-Only Correlation Matching

For each scale k, local patches centered on detected interest points go into the frequency domain, and we compute phase-only correlation as:

$$r_{k}(x,y)=F^{-1}\left(\frac{F_{s}F_{t}^{*}}{|F_{s}F_{t}^{*}|}\right)$$

where $F_{s}$ and $F_{t}$ are the Fourier transforms of the reference and target patches, $F_{t}^{*}$ is the complex conjugate of $F_{t}$ , and $F^{-1}$ denotes the inverse Fourier transform.

We then estimate the translational displacement from the location of the peak in the correlation surface. That peak corresponds to the best match between the two patches $\left(\Delta x_{k},\Delta y_{k})=arg\;{max}_{\left(x,y\right)}r_{k}(x,y\right)$ .

### 3.7. Multi-Scale Displacement Accumulation

The proposed multi-scale matching strategy follows a coarse-to-fine scheme for displacement estimation. For each interest point, we build a Gaussian pyramid with three resolution levels. At each level *k*, phase correlation gives us an initial displacement $\Delta_{k}$ . Then we refine it to sub-pixel accuracy using 1D parabolic [24].

Let k=2 be the coarsest level, k=1 the intermediate, and k=0 the finest. To obtain the final displacement, we scale contributions from all levels to the original resolution and accumulate them.

$$\Delta =\sum_{k=2}^{0}\Delta_{k}\cdot s_{k}\;\mathrm{with}\;s_{k}=2^{k}$$

We start at the coarsest level, compute the displacement, and scale it to the original image size. That result becomes the initial estimate for the next finer level, where it is refined. We repeat the same procedure until the finest level, and each stage progressively improves the final displacement accuracy.

### 3.8. Sub-Pixel Refinement

The pyramid-based phase correlation provides integer-pixel displacements, which are not sufficient for sub-pixel accurate applications. A refinement step is therefore introduced by estimating a correction term ϵ, leading to the final displacement $\Delta^{*}=\Delta +\epsilon$ . Refinement is applied locally on 16 × 16 patches and validated using a minimum correlation peak threshold of 0.25. Next, we apply a parabolic sub-pixel estimation to the correlation peak, following Foroosh et al. (2002) [24]. Subpixel refinement by 2D paraboloid fitting consists of locally modeling the correlation peak using a complete quadratic polynomial $f\left(x,y\right)=ax^{2}+by^{2}+cxy+dx+ey+f$ . From a 3 × 3 pixel window extracted around the correlation maximum, the six coefficients are estimated using least squares. Then we cancel the partial derivatives to obtain the optimal subpixel position, Δ*x* and Δ*y* corrections are typically within ±0.5 pixels. Because this method uses the local curvature of the peak, it performs better than simple linear approaches in terms of accuracy.

### 3.9. Candidate Correspondence Selection

Phase-only correlation gives us the estimated location in the target image for each interest point at scale k. Robustness and efficiency are improved by a three-step selection. First, we only search a circular neighborhood of radius R = 15 pixels around the predicted position. Second, we only keep candidate matches when the correlation peak max(*r*) > τ (τ = 0.25). Finally, to avoid ambiguous matches, the peak ratio $\frac{r_{\mathrm{peak}}}{r_{\mathrm{second}\;\mathrm{peak}}}>\gamma$ between the highest and second-highest must be greater than *γ* (1.5 ≤ *γ* ≤ 2.0). This is how we get reliable correspondences in VHR multi-temporal images. The thresholds τ and *γ* were selected empirically from preliminary experiments conducted on representative subsets of the datasets and were kept unchanged throughout all experiments. The correlation peak threshold was used to discard weak correspondences, whereas the peak ratio criterion was retained to preferentially select correspondences with a dominant correlation peak that is sufficiently distinct from secondary peaks.

### 3.10. Removal of Duplicate Correspondence Points

After matching, we filter duplicates to obtain unique correspondences between reference and target images. First, we group matches by rounded coordinates in the reference image. If several matches share the same spot, we keep only the one with the highest correlation score. Then we do the same for the target image, so each point links to just one correspondence.

This enforces a one-to-one matching constraint and cleans out ambiguous or redundant matches. Keeping only the most reliable correspondences improves geometric consistency in the registration process and reduces how much mismatches affect transformation estimation.

This enforces a one-to-one matching constraint and keeps only the best correspondence for each point. Matching ambiguity is reduced and transformation estimation is improved as a result.

### 3.11. Outlier Rejection Using RANSAC

Outliers in the initial correspondences are mainly caused by noise, repetitive patterns, or incorrect phase-correlation matches. We removed them using the Random Sample Consensus (RANSAC) algorithm. At each iteration, it selects a random subset of matches to estimate a similarity transformation. Then it computes reprojection errors for all correspondences. Matches with errors below a threshold are considered inliers, the others are rejected. After multiple iterations, the algorithm selects the model with the highest number of inliers.

In our implementation, RANSAC is applied globally after we merge all tile correspondences. We estimate a similarity transformation with a threshold of 0.5 pixel and run up to 5000 iterations. This happens after the refinement stage to make sure everything is globally consistent.

This process removes incorrect matches while keeping the reliable ones. It improves the final transformation accuracy and helps maintain sub-pixel precision, with most inliers showing registration errors below half a pixel.

### 3.12. Transformation Model Estimation

Using the refined correspondences, a global geometric transformation T is estimated to map points from the source to the target image, $T:(x,y)\to (x^{\prime },y^{\prime })$ . Depending on scene complexity, T can be modeled as an affine transformation for global linear distortions, a polynomial transformation for higher-order geometric deformations, or a Thin Plate Spline for non-rigid variations. The transformation parameters are estimated by minimizing the least-squares error between corresponding points:

$$\underset{T}{min}\sum_{i}∥T(P_{i}^{s})-P_{i}^{t}{∥}^{2}$$

where $P_{i}^{s}$ and $P_{i}^{t}$ denote corresponding point in the source and target images, respectively.

Based on the comparative evaluation in Rasmy et al. (2021) [21], we used a first-order polynomial transformation for the final registration step. It gave better results than the Thin Plate Spline (TPS) model on all tested datasets, both in terms of RMSE and computation time.

### 3.13. Evaluation Criteria

Root Mean Square Error (RMSE), the percentage of errors under 1 pixel and 0.5 pixel, Median Absolute Error (MedAE), and inlier ratio are used to measure accuracy, robustness, and reliability of the estimated correspondences (see Table 1).

## 4. Results and Discussion

We now report the results of our proposed registration method. The experiments used Pleiades and Sentinel images of the Fes region. We analyze the effects of patch size, windowing functions, multi-scale decomposition, and sub-pixel refinement on the final registration accuracy. All RMSE values are expressed in pixels unless otherwise specified. 

### 4.1. Data Descriptions

For the experiments, we used optical satellite imagery. The dataset includes two multi-temporal images acquired by Pleiades and Sentinel over the Fez region (see Table 2). Fez was chosen because it contains urban areas, agricultural land, and rugged terrain (elevations from 180 m to 837 m). These characteristics introduce geometric distortions, local scale variations, and significant radiometric and geometric differences between the two acquisition dates, making image registration particularly challenging. To evaluate our method on different kinds of terrain, eight image regions of 2000 × 2000 pixels were extracted and evenly distributed across the study area.

The registration accuracy was evaluated using manually selected Ground Control Points (GCPs) identified on the reference and sensed images. The sensed images used in the experiments were geometrically corrected prior to the evaluation. The GCPs were carefully selected on stable, well-defined features, such as road intersections, building corners, and other permanent landmarks, that could be unambiguously identified in both images. These control points served as the reference for the quantitative assessment of registration accuracy.

### 4.2. Influence of Patch Size and Corner Detector on Registration Performance

**Figure 2:**
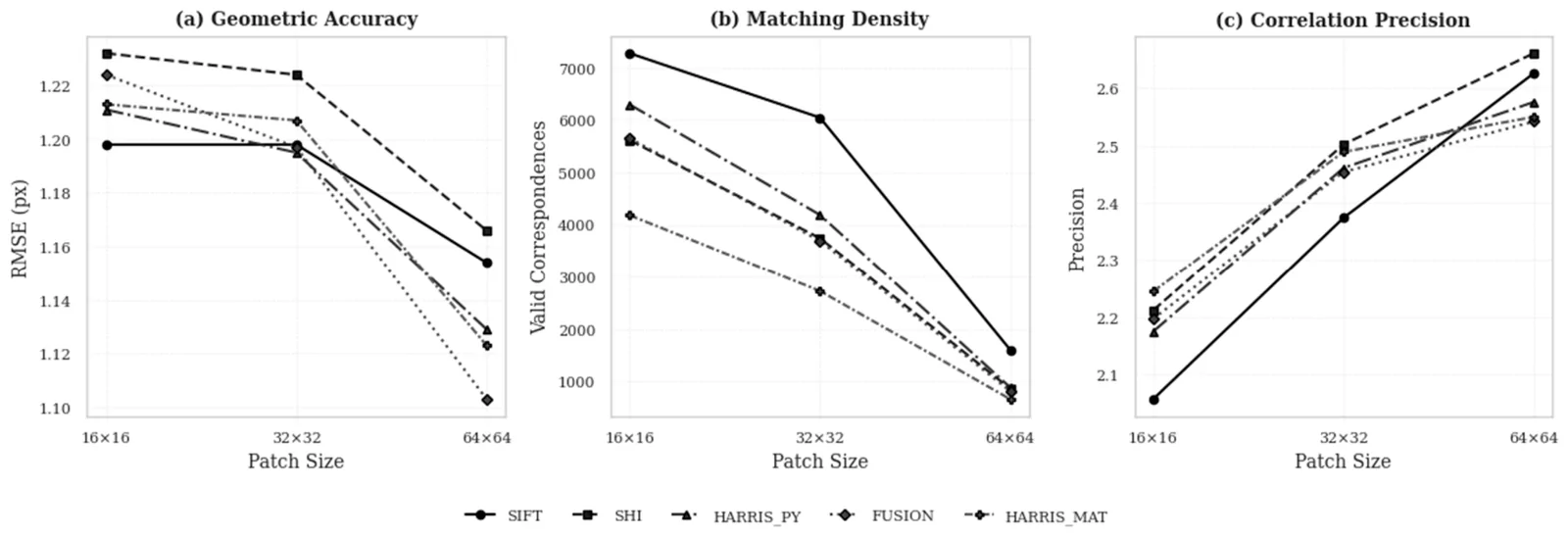
Performance comparison of keypoint detectors across patch sizes (px = pixel). The unit of RMSE is pixels.

Figure 2 compares different feature detectors and patch sizes for geometric accuracy and match quality. Registration performance depends on both components.

With 16 × 16 patches, all detectors produce many inliers. SIFT gives the most (7271 inliers) and shows the best repeatability. However, RMSE remains high (1.198 to 1.232 px). Small patches are sensitive to noise and local ambiguities. They also reduce geometric stability because local structures are often not distinctive enough. For the intermediate 32 × 32 patches, accuracy and robustness are better balanced. RMSE decreases slightly compared to 16 × 16. The inlier ratio increases to about 27% for SIFT and Harris-Py. With 64 × 64 patches, Fusion achieves the lowest RMSE (1.103 px), the highest sub-pixel rate (56% below 1 pixel), and a strong MedAE (0.936). Larger patches improve registration precision by capturing more contextual information, but they reduce the number of matches. SIFT still produces the highest number of inliers (47%) and high repeatability. However, Fusion gives the best balance between accuracy, stability, and efficiency for VHR registration.

We found that the coarse-to-fine matching strategy plays a key role in registration accuracy. A 16 × 16 pixel patch was chosen to keep enough points for reliable transformation estimation, since subpixel refinement and RANSAC come later.

### 4.3. Ablation Analysis of the Proposed Fusion Detector

Four configurations were used in an ablation study to examine the effect of progressively integrating the components of the proposed Fusion detector: (i) Shi–Tomasi only, (ii) FAST only, (iii) Shi–Tomasi + FAST, and (iv) a complete Fusion detector that combines Shi–Tomasi, FAST, edge detection, and probabilistic Hough line filtering. All configurations were evaluated using the same registration pipeline.

As shown in Table 3, Shi–Tomasi produced the largest number of inliers (267), whereas FAST achieved a lower RMSE (0.258 pixels) but with fewer inliers (79). Combining Shi–Tomasi and FAST increased the average correlation peak but did not improve the overall matching performance. Incorporating the Canny edge detector and probabilistic Hough line filtering further improved the quality of the retained correspondences, yielding the lowest RMSE (0.010 pixels), the lowest MedAE (0.001 pixels), and the highest inlier ratio (0.406). Although its average correlation peak was lower than those of FAST and Shi–Tomasi + FAST, these results suggest that the additional edge- and line-based filtering stages help retain more geometrically consistent correspondences, thereby improving subpixel registration accuracy.

### 4.4. Effect of Apodization Windows on Phase-Correlation-Based Registration

**Figure 3:**
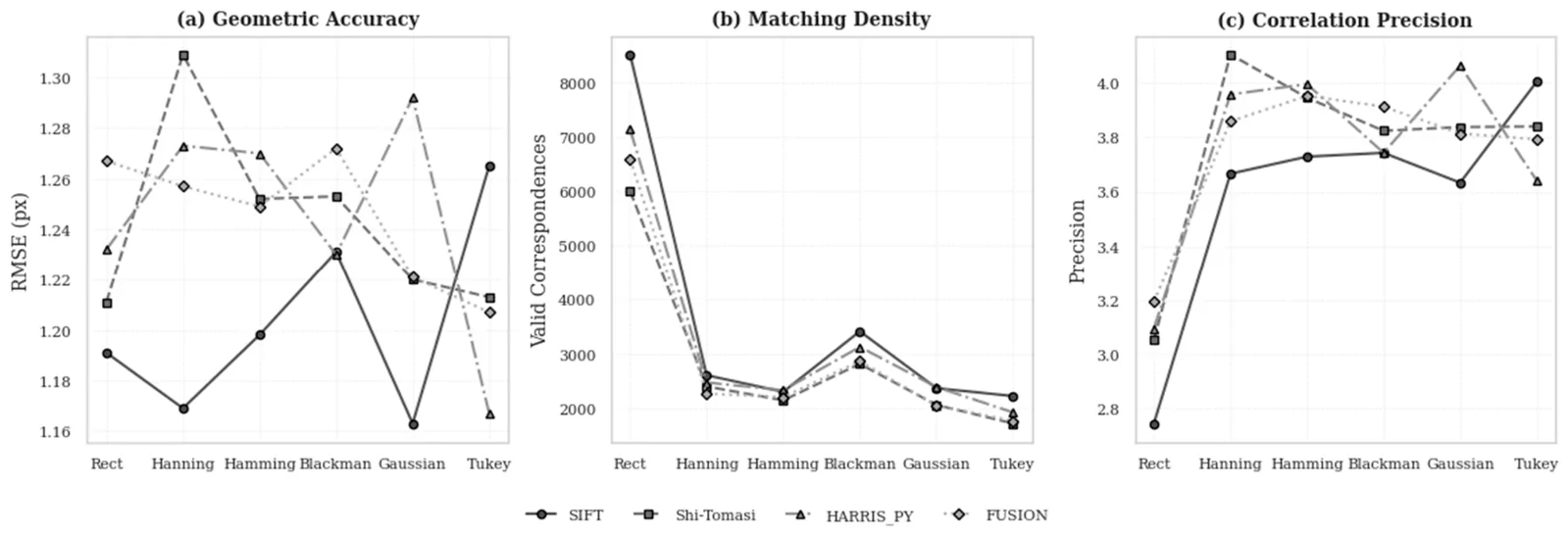
Precision and correspondence density across different apodization windows and feature detectors (px = pixel). The unit of RMSE is pixels.

Figure 3 shows that the influence of apodization on phase-correlation-based registration affects performance in complex ways. Classical theory says spectral windowing should improve registration accuracy by reducing spectral leakage and stabilizing the correlation response. However, the results show that in VHR remote sensing imagery, preserving high-frequency structural information can matter as much as aggressive boundary smoothing. The results therefore reveal a trade-off between spectral stabilization and the preservation of discriminative image content, which ultimately governs registration performance.

Non-apodized phase correlation is theoretically more sensitive to spectral leakage caused by boundary discontinuities. But the results show that preserving the original image signal often yields stronger correlation responses and competitive geometric accuracy. SIFT without apodization reaches a peak value of 0.482, higher than Gaussian (0.298), Tukey (0.294), Hanning (0.297), and Hamming (0.296). Harris-Py achieves an RMSE of 1.195 pixel, better than Hanning (1.273 px), Hamming (1.270 pixel), and Gaussian (1.292 px). Fusion also reaches high subpixel accuracy, with 47.43% below one pixel.

Gaussian apodization gives the best overall balance. It achieves the lowest RMSE (1.163 px) and highest subpixel ratio (49.54%) with SIFT. It stabilizes the spectrum. It preserves sufficient texture. Rectangular windows produce the highest inliers (8505 for SIFT). But they reduce subpixel accuracy because of spectral leakage. Tukey windows also perform well and preserve the central region while attenuating boundaries. Hanning and Hamming improve subpixel accuracy but strongly reduce repeatability and match density. Blackman performs worst because it suppresses too much high-frequency information.

In VHR imagery, preserving high-frequency structure is essential for accurate registration. Strong tapering can degrade performance by removing discriminative information. But moderate or adaptive windows give a better balance between stability and structure preservation.

### 4.5. Influence of Multi-Scale Decomposition on Phase-Correlation Accuracy

**Figure 4:**
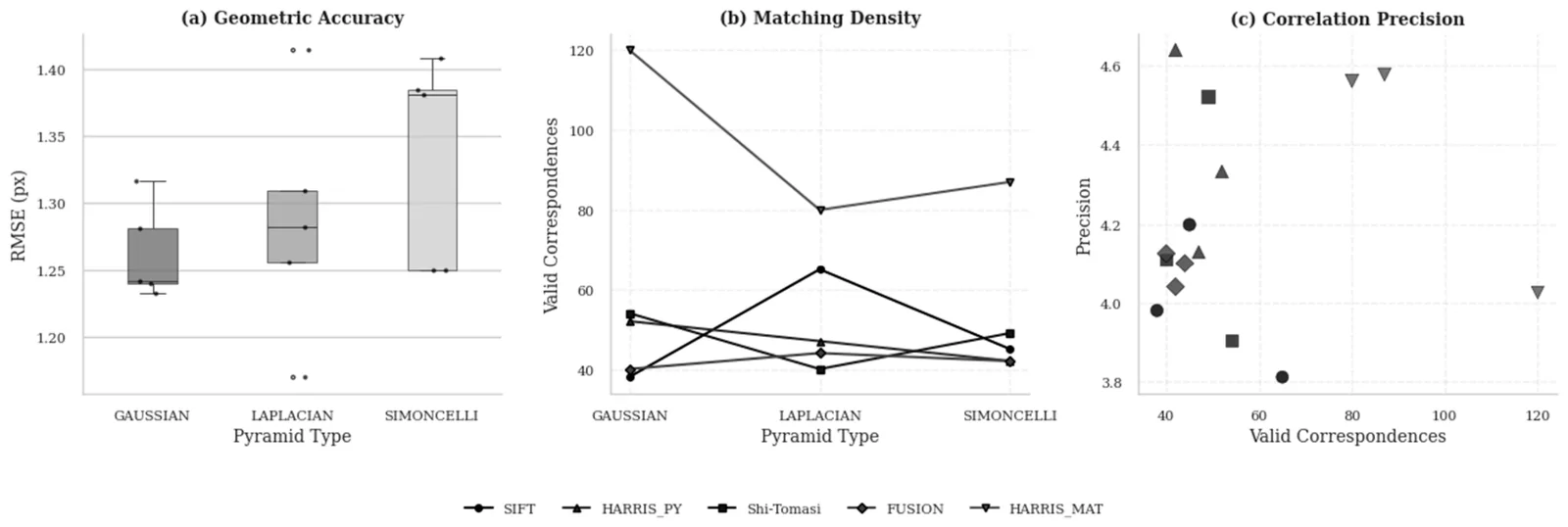
Shows multi-scale phase correlation performance with different pyramid decomposition methods (px = pixel). RMSE is measured in pixels.

To study how multi-scale decomposition affects registration and find the best approach, we applied Simoncelli, Gaussian, and Laplacian pyramids to source and target images around Harris interest points (see Figure 4). The correct correspondence rate is a key criterion for evaluating each method. We look at how each decomposition represents image information across scales, from fine details to global structure, for reliable matching. Pyramid strategies strongly affect phase-correlation-based registration, with clear differences in accuracy and stability.

The Gaussian pyramid gives the most stable and balanced results. It progressively smooths and downsamples the image while keeping dominant low-frequency structures. This improves coarse-to-fine consistency and reduces sensitivity to noise. The best RMSE values come from Gaussian decomposition: SIFT with Gaussian apodization (1.163 px) and SIFT with Tukey apodization (1.114 px). It also keeps computational cost moderate and subpixel behavior stable, a good balance between spectral stability and structural preservation.

The Laplacian pyramid preserves stronger edge and high-frequency information by encoding band-pass residuals between scales. This increases correspondence density and improves robustness in textured VHR images, especially with rectangular apodization. For example, with rectangular apodization, SIFT reaches 606 correspondences and a high inlier ratio. But this focus on high frequencies can increase sensitivity to noise and slightly reduce geometric precision.

The Simoncelli pyramid shows the most variable behavior. It uses steerable filters that give richer directional and spectral representations, but this raises computational cost and reduces stability across configurations. In some cases, like Harris-Py with Tukey apodization (1.142 px), the RMSE is low, which shows strong dependence on the detector-window combination.

So, Gaussian pyramids give the most stable performance. Laplacian pyramids improve local detail and match density. Simoncelli pyramids offer richer representations, but need careful tuning and more computation.

### 4.6. Enhanced Subpixel Registration Accuracy

We evaluate several approaches grouped into three types: spatial methods (paraboloid fitting [24], sinc interpolation [45], Gaussian peak fitting [22,25], and centered-difference interpolation [24]), frequency-based methods (Stone [27], FFT upsampling [46]), and optimization methods (Nelder–Mead [47], TPSS gradient [48]).

**Figure 5:**
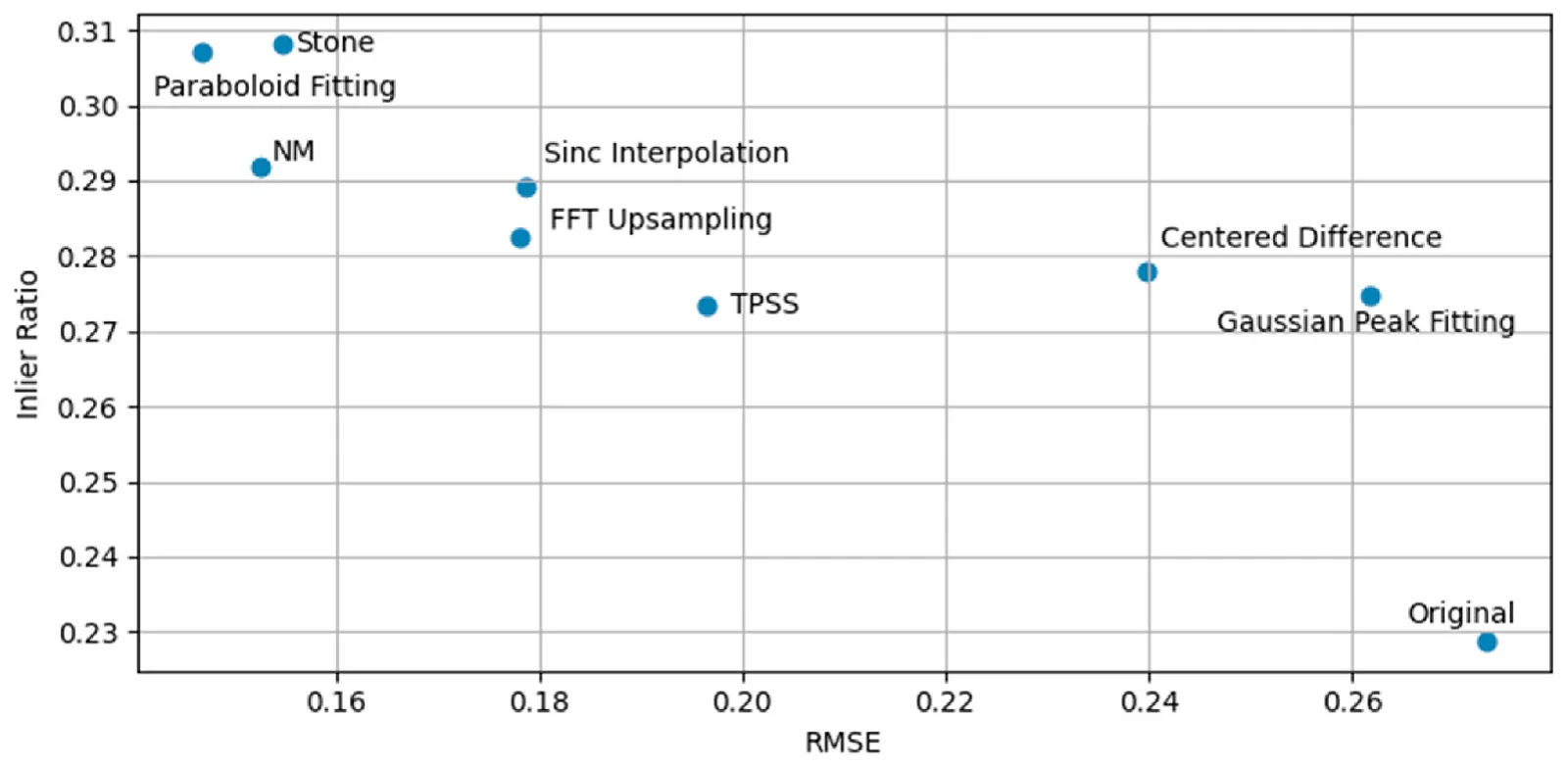
RMSE and inlier ratio metrics for the subpixel methods.

**Figure 6:**
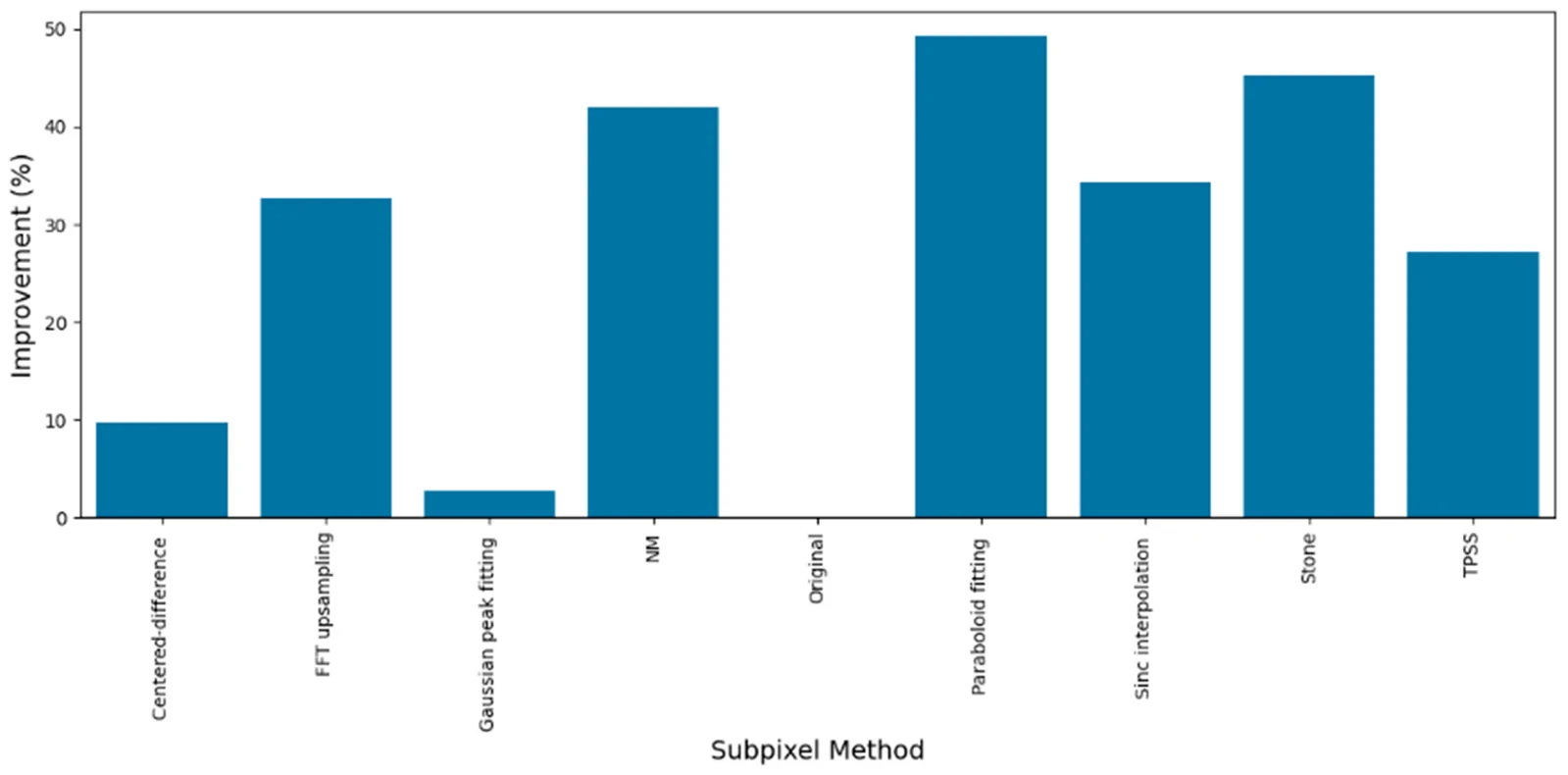
Improvement rate of each subpixel method (px = pixel).

Figure 5 and Figure 6 show that subpixel refinement performance depends on both the refinement strategy and the corner detector. Paraboloid fitting and TPSS emerge as the most reliable refinement methods. In particular, paraboloid fitting combined with Fusion yields minimal registration error (RMSE = 0.010 px, 96.3% improvement) and high inlier ratios (40.6%), confirming its suitability for accurate VHR image alignment. Fusion and Harris-Py achieve higher matching quality, with lower RMSE and higher inlier ratios. These findings confirm that performance depends on the joint optimization of detector–refinement combinations.

### 4.7. Effect of Relief on Phase Matching

The influence of topography on phase-correlation-based matching was evaluated for three slope classes: gentle terrain (5–15%), hilly terrain (15–30%), and mountainous terrain (30–50%). Accuracy decreases gradually as slope increases. Accuracy decreases gradually as slope increases (Table 4). In hilly terrain, errors become larger as relief effects and residual geometric distortions reduce the similarity between corresponding patches. The highest errors occur in mountainous areas, where steep slopes introduce stronger geometric distortions, shadow effects, occlusions, and parallax displacements, making correspondence estimation more difficult. Overall, the increase in RMSE remains moderate compared with the rise in terrain complexity, indicating that the proposed multi-scale framework maintains robust performance as terrain becomes more complex. However, in mountainous terrain combined with large viewing-angle differences, the achievable registration accuracy reaches a practical limit. Under these conditions, the registration error is primarily governed by the imaging geometry rather than by the registration algorithm itself. Consequently, further improvements are unlikely to be achieved through refinement of the proposed multi-scale framework alone and would instead require accurate digital elevation models or more advanced geometric correction models.

### 4.8. Validation of the Proposed Registration Method

We compared our method to three others: LoFTR, SIFT-based method and our own baseline approach. For SIFT, we test both the standard feature-based pipeline and a version combined with our phase-correlation strategy. This helps clarify the role of the matching stage in the overall pipeline. The estimated misregistration, measured on a set of independent check points, was found using first-order polynomial transformations, in each of the x and y dimensions (see Table 5).

Before registration, the Pleiades imagery shows noticeable geometric misalignment, with errors of 0.968 m in x and 1.968 m in y for Pleiades imagery, showing the need for accurate registration.

All methods were shown to significantly improve the accuracy. The original Harris-based method already reaches sub-pixel accuracy (RMSE_x_ = 0.301 m, RMSE_γ_ = 0.304 m). With the proposed multi-scale refinement, our approach achieves the best horizontal accuracy (RMSE_x_ = 0.248 m), which confirm the benefit of coarse-to-fine refinement for stable feature localization.

Our approach with SIFT detector performed similarly in x-direction (RMSE_x_ = 0.260 m). But worse in y-direction (RMSE_γ_ = 0.637 m). The standard SIFT approach shows the weakest results (RMSE_x_ = 0.516 m, RMSE_γ_ = 0.679 m), confirming that descriptor matching alone is insufficient for high-precision registration.

LoFTR behaves differently. It has higher error in x (RMSE_x_ = 0.402 m) but the best vertical accuracy (RMSE_γ_ = 0.216 m). This reflects its ability to capture global context. But its performance is less balanced across directions, and some correspondences fall outside the image domain. This means the model extrapolates matches beyond valid spatial limits, so it becomes unstable in some regions.

Our approach gives the most stable and balanced accuracy. But LoFTR shows a strong ability to handle certain directional deformations. So, combining the geometric stability of multi-scale approaches with the contextual capabilities of deep learning-based matching methods looks promising for image registration.

## 5. Conclusions

This work presents a unified multi-scale framework for VHR image registration that combines phase correlation, Harris corner detection, and sub-pixel refinement. The contribution lies not only in the proposed pipeline but also in the systematic analysis of how each component influences registration accuracy and robustness under different imaging and terrain conditions.

Experiments are conducted on multi-temporal Pleiades and Sentinel images over urban and rural areas. Performance depends on a clear hierarchy of factors. The most important is the multi-scale representation. Gaussian pyramids give the most stable behavior across scales by preserving low-frequency structures and ensuring reliable coarse-to-fine alignment. Patch size also plays a key role, with 64 × 64 giving the best compromise between accuracy, robustness, and computational cost.

At the next level, detectors and spectral processing affect correspondence quality. SIFT achieves the highest accuracy, while Harris-based detectors offer a better trade-off between speed and performance. Fusion gives stable results on limited hardware with a high sub-pixel success rate. Windowing can improve stability in some cases: Gaussian and Tukey reduce spectral instability while preserving useful structure, although apodization is not always necessary when the signal is already stable.

Sub-pixel refinement further improves localization accuracy. Paraboloid fitting is the most consistent method, reaching errors as low as 0.010 pixel. FFT upsampling and TPSS methods perform well but depend more on detector quality. Refinement performance strongly depends on the quality of the correlation peak produced in earlier stages.

The proposed framework provides reliable registration across diverse terrain conditions. Nevertheless, under severe topographic relief and large viewing-angle differences, registration accuracy becomes primarily limited by the imaging geometry, indicating that further algorithmic refinement alone is unlikely to produce significant improvements.

Compared with the SIFT-based method and LoFTR, our method is more consistent in challenging conditions and remains competitive with deep learning approaches in terms of accuracy and robustness. But it still struggles in low-texture areas and under strong non-linear distortions. Future work will focus on hybrid approaches that combine our multi-scale phase-correlation framework with learning-based methods to improve robustness and generalization.

## Figures and Tables

**Table 1:** Evaluation metrics for registration quality assessment.

Metric	Formula	Definition/Purpose
RMSE	$\sqrt{\frac{1}{n}\sum ∥x_{i}^{\prime }-T(x_{i}){∥}^{2}}$	Global geometric accuracy: n denotes the number of validation points, $x_{i}^{\prime }$ the reference position, and $T\left(x_{i}\right)$ the estimated position after registration.
sub-pixel accuracy % < *δ* pixel	$\frac{1}{n}\sum I(∥x_{i}^{\prime }-T(x_{i})∥<$ *δ*) × 100	Sub-pixel accuracy: *δ* ∈{1,0.5}
Inlier Ratio	$(N_{inliers}/N_{matches})\times 100$	Robustness to false matches: $N_{inliers}$ denotes the number of inlier correspondences, and $N_{matches}$ the total number of initial correspondences.
MedAE	$median(∥x_{i}^{\prime }-T(x_{i})∥)$	Robust error measure
Improvement	$100\times \frac{{\mathrm{RMSE}}_{\mathrm{org}}-{\mathrm{RMSE}}_{\mathrm{ref}}}{{\mathrm{RMSE}}_{\mathrm{org}}}$	Registration accuracy improvement: ${\mathrm{RMSE}}_{\mathrm{org}}$ denotes RMSE before subpixel refinement, and ${\mathrm{RMSE}}_{\mathrm{ref}}$ the RMSE after subpixel refinement.

**Table 2:** Summarizes the parameters for the Pleiades (1) and Sentinel-2 (2) datasets.

Datasets		Image Size (Pixels)	Year	Resolution (m)	Incidence Angles (°)	Solar Elevation Angle (°)	Solar Azimuth Angle (°)
(1)	Reference image	37,430 × 42,068	2013	0.5	18	31	161
	Target image	25,855 × 38,808	2014	0.5	−17	30	160
(2)	Reference image	10,980 × 10,980	2019	10	8.1	70.8	124.5
	Target image	10,980 × 10,980	2021	10	8.1	68.8	129.7

**Table 3:** Ablation study results for the proposed Fusion detector.

Detector	RMSE	RMSE x	RMSE y	MedAE	% <1 Pixel	% <0.5 Pixel	Inlier Ratio	Correlation Peak	Inliers
Shi–Tomasi	0.309	0.220	0.216	0.320	100	99	0.184	0.399	267
FAST	0.258	0.196	0.168	0.175	100	95	0.095	0.753	79
Shi–Tomasi + FAST	0.266	0.158	0.214	0.205	100	98	0.090	0.827	59
Fusion	0.010	0.006	0.008	0.001	100	100	0.406	0.457	81

**Table 4:** Influence of terrain slope on phase-correlation matching accuracy. The unit of RMSE is pixels.

Slope Class	Slope (%)	RMSEx	RMSEy	Inliers Ratio
Gentle terrain	5–15	0.040	0.050	2.68
Hilly terrain	15–30	0.154	0.120	1.03
Mountainous terrain	30–50	0.179	0.143	0.47

**Table 5:** Horizontal and vertical accuracy of the target image for different registration approaches. (1) Pleiades imagery and (2) Sentinel imagery. The unit of RMSE is meters.

Methods	RMSEx	RMSEy	RMSEx	RMSEy
	(1)		(2)	
Target image	0.968	1.968	1.585	10.636
Original approach-Harris	0.301	0.304	3.182	3.147
Our approach	0.248	0.319	2.120	2.216
Our approach—SIFT	0.260	0.637	3.275	2.452
SIFT	0.516	0.679	5.028	5.148
LoFTR	0.402	0.216	4.85212	3.707

## Data Availability

The data used in this study include freely available Sentinel satellite imagery and very high-resolution Pleiades images provided for research purposes. The Sentinel data and derived products are publicly available. The Pleiades imagery is subject to licensing restrictions and cannot be publicly shared.
